# Physicians’ knowledge of and adherence to improving oral health

**DOI:** 10.1186/1471-2458-12-855

**Published:** 2012-10-09

**Authors:** Sepideh Rabiei, Simin Z Mohebbi, Kristiina Patja, Jorma I Virtanen

**Affiliations:** 1 Community Oral Health Department, Tehran University of Medical Sciences, P.O. Box 1439955991, Tehran, Iran; 2 Department of Oral Public Health, University of Helsinki, P.O. Box 41, FI-00014, Helsinki, Finland; 3 Pro Medico, Association for Medical Continuous Professional Development in Finland, P.O. Box 49, 00501, Helsinki, Finland; 4 Department of Public Health, University of Helsinki, P.O. Box 41, 00014, Helsinki, Finland; 5 Department of Community Dentistry, University of Oulu, P.O. Box 5281, 90014, Oulu, Finland

**Keywords:** Attitude, Knowledge, Physician, Primary care, Dentistry

## Abstract

**Background:**

Integration of oral health promotion into general health care has been highly recommended by the World Health Organization. Primary-care physicians can as part of their general health care promote and contribute to improved oral health care. Our aim was to investigate primary-care physicians’ knowledge of oral health, their attitudes toward delivering oral health care (OHC), and their willingness to obtain more education in this field.

**Methods:**

We conducted a cross-sectional survey of all primary-care physicians working in the public health centers of Tehran city. An anonymous self-administered questionnaire queried their knowledge in pediatric- and general medicine-related areas of dentistry, providing knowledge scores to be calculated for three domains. The physicians’ attitudes toward OHC and willingness to pursue continuous education underwent evaluation with statements utilizing a 5-point Likert scale. Totally, 220 physicians took part in the survey (response rate: 92%). Chi-square test, linear and logistic regression, and t-test served for statistical analyses.

**Results:**

The physicians’ knowledge score was significantly lower in the pediatric domain than in the dental and medical domains (p < 0.001). The number of physicians answering correctly to the pediatric questions was less than 40%. Almost all physicians (95%) reported it necessary for a physician to know about OHC and admitted (78%) that physicians’ general knowledge in this field is inadequate. Further, 77% of the physicians expressed a will to implement preventive oral health activities in their practice, and almost two-thirds (62%) of them showed a willingness to pursue further education about OHC. Those with higher knowledge scores had a greater willingness to deliver oral health care to their patients.

**Conclusions:**

Physicians’ lack of knowledge of OHC and their generally positive attitudes toward it revealed a great need for planning of a continuous medical education program in primary care.

## Background

Oral health contributes to morbidity and mortality throughout one’s lifespan [1]. In childhood, parental health behaviors influence children’s oral health through diet and dental care, which can emerge as dental diseases, mainly caries, increasing the risk for multiple chronic diseases [2]. In adolescence, preventing unhealthy behavior such as use of tobacco and alcohol, links oral health to medical care and prevention. In addition several oral diseases have important side effects on general health, while systemic conditions may show a mutual influence on oral health. Therefore, oral health care needs to be addressed by a multi-professional approach and should be integrated into comprehensive health-promoting strategies and practices [2-4]. Currently, such integration has been evident not only in public health policies, but also in chronic care models treating diabetes or depression [5]. In addition, the common risk factor approach for chronic diseases calls for multi-professional collaboration [6,7]. Thus, oral health promotion is needed within health care practices of physicians and nurses [8,9]. Comprehensive approaches are certainly even more important and efficient in health care service systems with less-developed public dental care.

Previous studies such as one from India have shown that primary-care medical providers can play an important role in helping individuals gain access to oral health care (OHC) in developing countries [10] and to introducing successful preventive measures [11-13]. In primary care, physicians meet children and their families regularly in child-health clinics, with excellent opportunities to promote oral health [14]. The effectiveness of a medical-dental partnership has been apparent especially among lower socio-economic groups at risk for oral diseases [15,16]. Obstacles to physicians’ attending to oral health prevention can relate to their knowledge, work environment, and attitudes. Medical curricula may include only limited information on OHC [15], and recent studies reveal that a high proportion of physicians working in pediatric health care lack sufficient knowledge on prevention of dental decay among children [11,17,18].

A need thus exists to develop sustainable OHC practices for primary care physicians, especially in countries with developing health care systems. This requires developing a full range of competencies: a set of skills and the improved knowledge and attitudes necessary to enable physicians to support both the broad practice of public health and in this case, oral health [19]. Not much research on general physicians’ knowledge of and adherence to OHC in the Eastern Mediterranean region is available; some studies have, however, shown low levels of OHC knowledge, attitudes, and practice among physicians [20,21]. Our aim was to study OHC knowledge among primary care physicians as well as their attitudes in terms of their perceived competence to deliver OHC and willingness to attend OHC training.

## Methods

We conducted a cross-sectional survey by means of a self-administered questionnaire. The survey was voluntary, with no identifiable information such as participants’ or centers’ name collected. The Ethics Committee of Tehran University of Medical Sciences approved the survey. The target population comprised all 241 physicians working in the public health centers of Tehran city in April 2011 who were eligible for the study. District Health Centers (DHC) agreed to distribute self-administered questionnaires to physicians, along with a pack containing a toothbrush and dental floss as a gift. Responses were anonymous. In total, 220 physicians returned completed questionnaires, for a response rate of 92% (Table 1).

**Table 1:** Distribution of physicians (n = 220) working in public health centers according to background characteristics in Tehran, Iran

	**Gender**						
	**Male**	**%**	**Female**	**%**	**Total**	**%**	**p-value**
Age^*^							
Mean (SD)	44.1 (7.7)		37.5 (6.7)		39.4 (8)		<0.001*
**Working Area**							
Affluent	20	33	52	33	72	33	0.900
Non-affluent	41	67	104	67	145	67	
**Working Sector**							
Public	15	27	122	84	137	69	<0.001
Public+Private	40	73	23	16	63	31	
**Total**	61	100	156	100	217	100	

^*^ Age was given by 209 individuals.

Statistical analyses by T-test and Chi square test.

Tehran has seven District Health Centers (DHC) with various functions including family health, environmental health, and dental health, and a medical education unit which took the responsibility of distributing the questionnaires in this study. Each DHC supervises 15 to 30 public health centers with a various number of physicians in each unit.

### Questionnaire and variables

The questionnaire of the study was developed based on previous validated surveys [16,18,22] with minor modifications. First, the questionnaire was assessed for content validity by experts in dental public health for relevancy and clearness. Then, a pilot study was conducted among physicians (n=30) working in the public health centers of Qazvin, a city near Tehran. Based on the pilot study (test/re-test at a two-week interval) and the participants’ opinion on the clarity of the questions, the questionnaire was slightly revised, and we omitted two questions with an actual agreement score under 70%.

Socio-demographics included the respondents' age, gender, their working profile (public only/public and private practice) and region (affluent/non-affluent).

The questionnaire contained statements on knowledge, attitude, and willingness for OHC education. The knowledge category included three domains: a) pediatric dentistry knowledge (12 questions), b) general dental knowledge (9 questions), and c) dentistry-related medical knowledge (13 questions). In the pediatric dentistry domain, questions included the timing of tooth eruption, the time/age to begin tooth cleaning and brushing for children and usage of fluoride for them, transmission of the bacteria that causes dental decay, the effects of pacifier sucking and mouth breathing, and the advantages of sealant therapy. The dental domain included questions on the first signs of dental decay and its etiology, the effects of fluoride and xylitol, the best time to refer a pregnant woman for a dental procedure, and the main causes of periodontal diseases. The medical domain consisted of questions on relations between systemic and periodontal diseases, drugs increasing risk for dental caries, and lesions in the oral cavity with need for biopsy. The responses were on the 5-point Likert scale with response alternatives ranging from "strongly agree" to ''strongly disagree,” including “don’t know.” In addition, we included four knowledge questions with multiple-choice answers.

Similarly, attitudes were measured with two statements on their opinion about the necessity of having OHC information and a request for the physicians to assess their competence in oral health. Their willingness was measured similarly by two statements on readiness to obtain more education and willingness to implement OHC activities.

### Data analyses

We dichotomized answers to the knowledge and attitude questions with a score of one for correct/willing/positive answers, and 0 for false/unwilling/negative and don’t-know answers. Thereafter, we calculated a total score for total knowledge (theoretical sum score: 34) and for its three domains: pediatric dental (theoretical sum score: 12) general dental (theoretical sum score: 9) and medical (theoretical sum score: 13) according to socio-demographic background factors.

Evaluation of statistical significance of the differences between subgroups included the independent samples t-test for comparison of mean values and the Chi-square test for frequencies. To compare the domains, the scores were standardized by calculating the percentage of correct answers inside domains. Statistical significances between the standardized mean scores were evaluated using analysis of variance with repeated measurements and paired samples t-test.

Linear Regression Models were used to investigate functional relationships associated with physicians’ knowledge scores, controlling for background characteristics. Logistic regression models served for the multivariable assessment of factors related to the willingness. The corresponding odds ratios (OR) and their 95% confidence intervals (95% CI) were determined. Goodness of fit was assessed by means of the R square (0.1 < R^2^ < 0.2) and Hosmer and Lemeshow tests (p = 0.67).

## Results

The distribution of primary care physicians working in the public health centers according to their background characteristics is shown in Table 1; this is representative of primary care in Tehran. A clear majority of the physicians were female (70%), and their mean age was lower than that of male physicians (37.5 vs. 44.1; p < 0.001). Most women were working in the public sector only, whereas most men did additional work in the private sector as well (p < 0.001).

**Figure 1:**
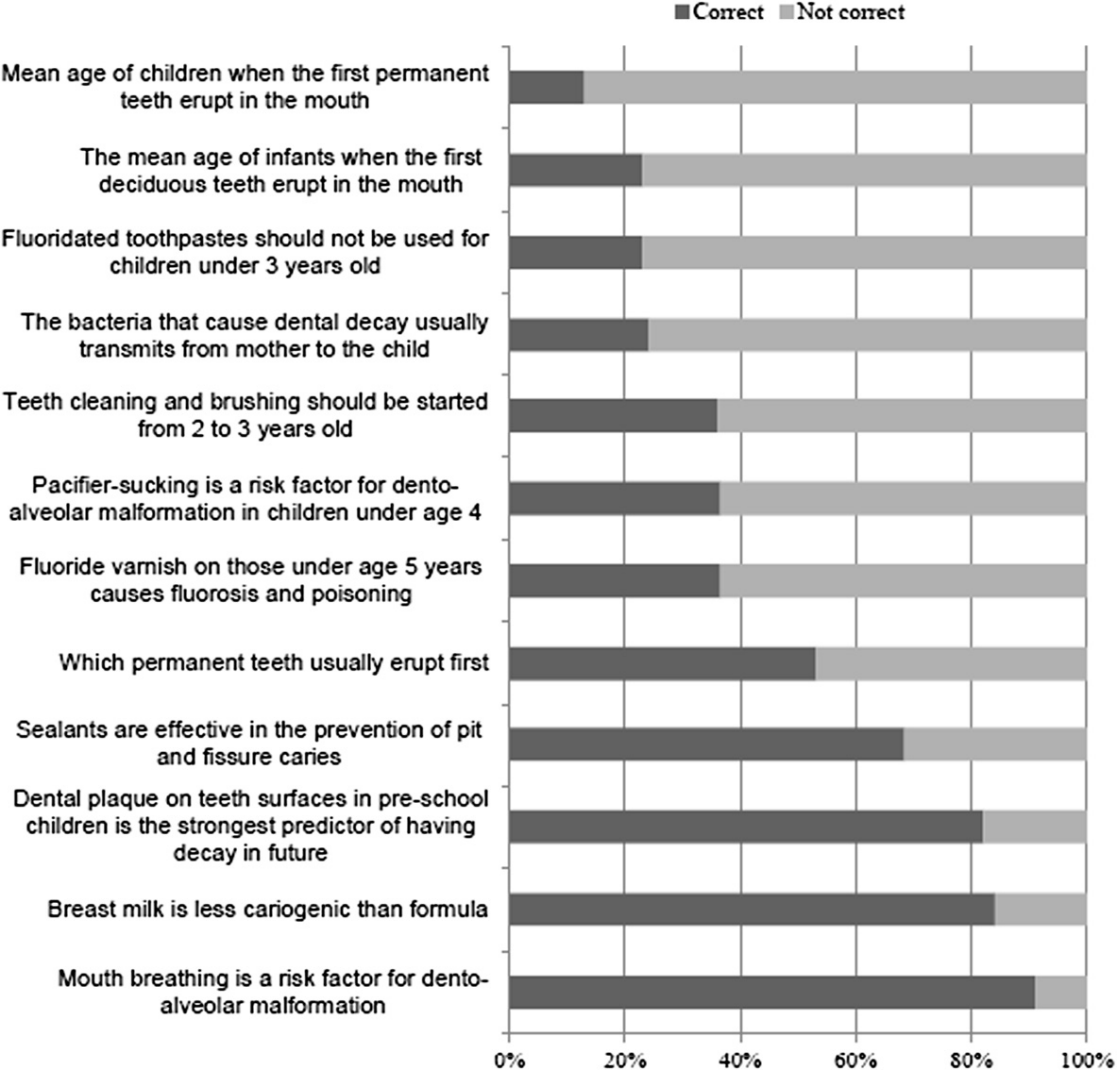
**Physicians’ answers to pediatric dentistry questions.** Detailed legend: The percentage of physicians’ (n = 220) correct answers to pediatric dentistry questions.

**Figure 2:**
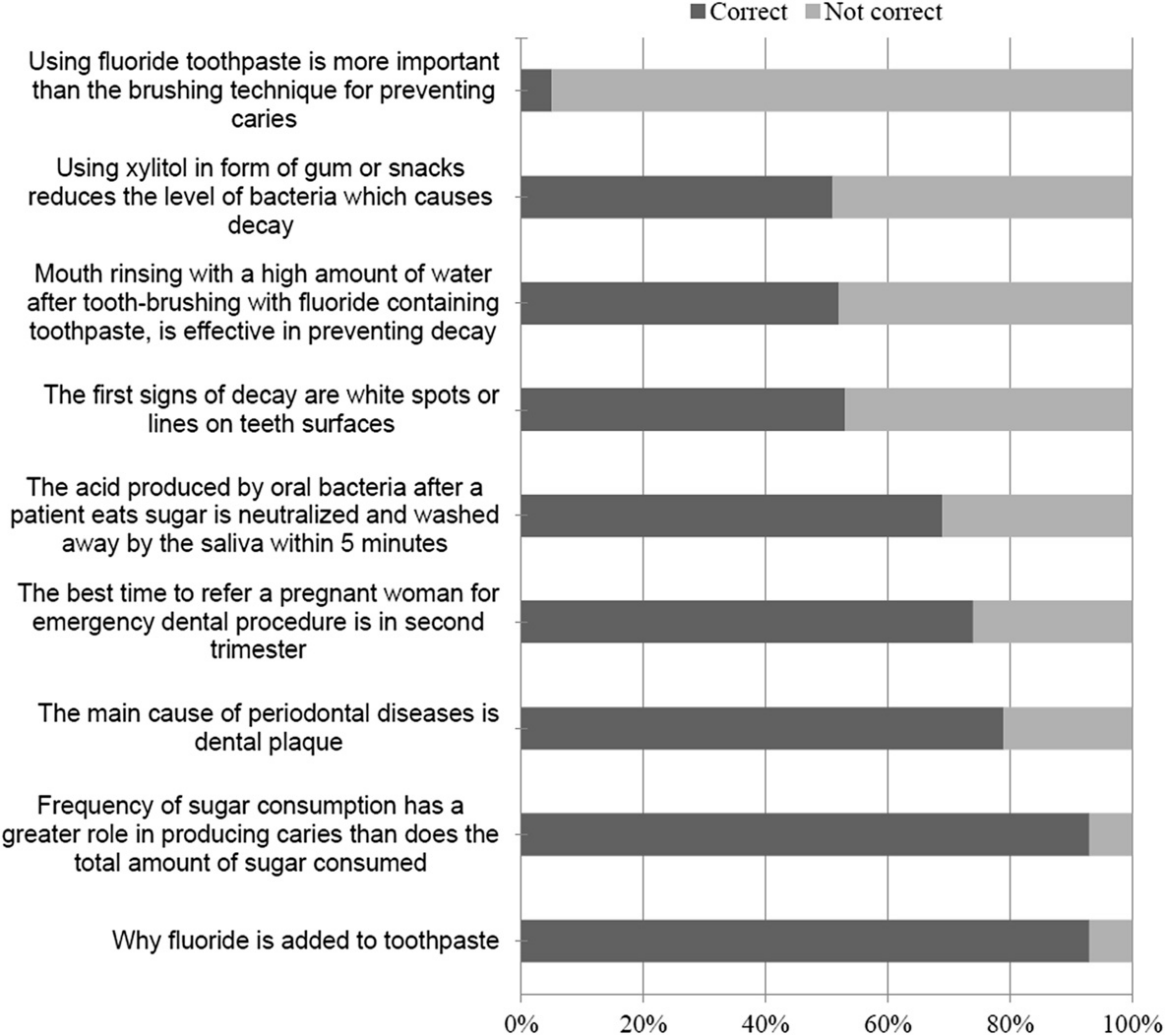
**Physicians’ answers to general dentistry questions.** Detailed legend: The percentage of physicians’ (n = 220) correct answers to general dentistry questions.

**Figure 3:**
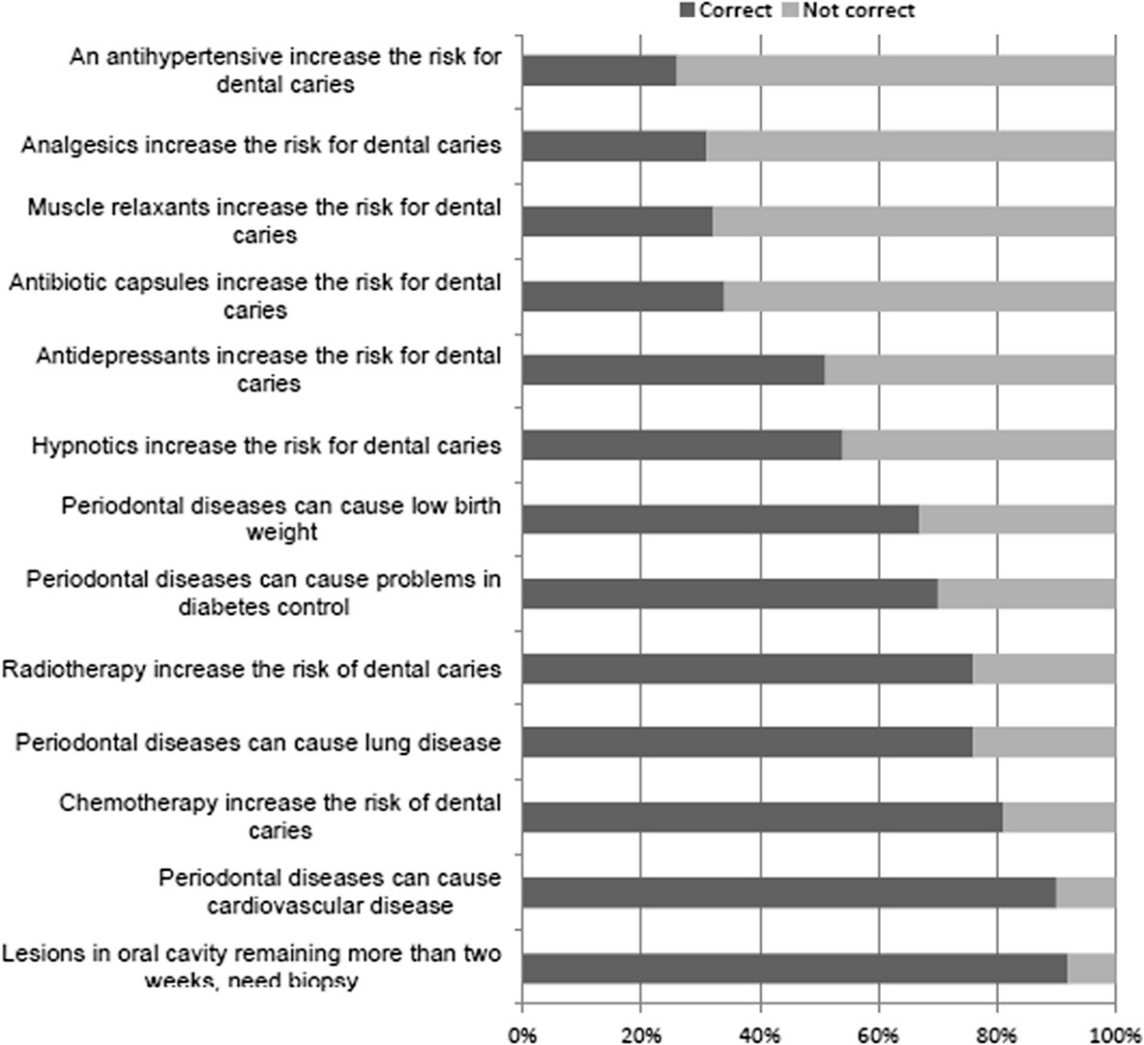
**Physicians’ answers to dentistry-related medical questions.** Detailed legend: The percentage of physicians’ (n = 220) correct answers to dentistry-related medical questions.

The knowledge scores of the physicians in oral health care according to background are shown in Table 2. The standardized mean score for the pediatric dentistry domain (45.0%, SD: 14.2) was significantly lower than for the dental (63.3%, SD: 16.8) and medical domains (56.2%, SD: 20.3) (p < 0.001). The number of physicians answering correctly was under 40% for seven pediatric dentistry questions out of twelve (Figure 1). The number of physicians answering correctly was under 40% for one dental domain question (Figure 2) and for four medical domain questions (Figure 3). Physicians working in both private and public sectors had significantly higher knowledge scores (p < 0.05) in all categories except when the pediatric dentistry domain was compared to that of physicians working in the public sector only. The older physicians' dental knowledge score was significantly higher than their younger colleagues’ (p < 0.05).

**Table 2:** Knowledge scores (mean, SD) in the three domains among the physicians (n = 220) by their background characteristics

	**Pediatric knowledge**	**Dental knowledge**	**Medical knowledge**	**Total knowledge**
	**Mean (SD)**	**Mean (SD)**	**Mean (SD)**	**Mean (SD)**
**Gender**				
Female	5.5 (1.7)	5.6 (1.5)	7.2 (2.6)	18.2 (4.6)
Male	5.1 (1.6)	5.7 (1.5)	7.4 (2.5)	18.1 (4.1)
p-value	0.180	0.939	0.741	0.934
**Age**				
24-41-year-olds	5.3 (1.9)	5.4 (1.6)	7.2 (2.7)	17.7 (5.0)
42-59-year-olds	5.5 (1.4)	5.9 (1.2)	7.3 (2.6)	18.6 (3.4)
p-value	0.423	0.010	0.959	0.149
**Working Region**				
Affluent	5.5 (1.5)	5.9 (1.4)	7.4 (2.7)	18.6 (4.2)
Non-Affluent	5.4 (1.7)	5.6 (1.5)	7.3 (2.5)	18.0 (4.6)
p-value	0.723	0.196	0.761	0.340
**Working profile**				
Public	5.3 (1.8)	5.6 (1.5)	7.0 (2.5)	17.6 (4.5)
Public+Private	5.6 (1.4)	6.0 (1.1)	8.0 (2.6)	19.5 (3.8)
p-value	0.395	0.018	0.012	0.005
**Total Score**	5.4 (1.7)	5.7 (1.5)	7.3 (2.6)	18.2 (4.5)

Statistical analyses by T-test.

Most of the physicians (95%) believed that it is necessary for a physician to know about OHC, and a clear majority (78%) admitted that physicians’ general knowledge about it is insufficient. In line with this, more than half (62%) showed a willingness to gain more information about OHC, and 77% of them expressed the will to implement preventive oral health activities in their practice. The physicians with higher knowledge scores showed greater willingness to deliver OHC for their patients compared to those with lower scores (p = 0.003). No gender or age differences were evident in these figures.

The linear regression model, controlling for background factors, endorsed the result that the working profile of the physicians had a significant effect on their knowledge scores, with those physicians working in both public and private sectors having higher scores (p < 0.05) in all categories except the pediatric part (Table 3). It also showed that females had generally higher scores in the pediatric domain than did the men (p < 0.05).

**Table 3:** Factors associated with physicians’ (n = 220) higher knowledge scores controlling for background characteristics as shown by Linear Regression Models

**Model**	**Unstandardized coefficients**		**Standardized coefficients**	**t**	**p-value**
	**B**	**Std. error**	**Beta**		
**Total knowledge score**					
Gender	−1.360	0.846	−0.138	−1.608	0.110
Age	0.525	0.669	0.059	0.784	0.434
Working profile	2.522	0.808	0.266	3.120	0.002
Working region	−0.468	0.687	−0.049	−0.682	0.496
**Medical knowledge score**					
Gender	−0.399	0.507	−0.068	−0.787	0.432
Age	−0.220	0.400	−0.042	−0.550	0.583
Working profile	1.318	0.484	0.234	2.722	0.007
Working region	−0.257	0.409	−0.046	−0.629	0.530
**Pediatric knowledge score**					
Gender	−0.655	0.330	−0.173	−1.986	0.048
Age	0.206	0.260	0.060	0.792	0.429
Working profile	0.501	0.315	0.138	1.592	0.113
Working region	−0.068	0.266	−0.019	−0.255	0.799
**Dental knowledge score**					
Gender	−0.311	0.317	−0.084	−0.979	0.329
Age	0.522	0.251	0.156	2.082	0.039
Working profile	0.700	0.303	0.196	2.311	0.022
Working region	−0.169	0.257	−0.047	−0.655	0.513

All variables were used in their continuous form except for gender, Working profile and working region.

Gender: 1 =female, 2=male.

Working profile: 0= only public sectors, 1= public and private sectors.

Working region: 1= affluent region, 2= non-affluent region.

t: T-test.

0.1<R square< 0.2.

Regarding the physicians’ willingness to deliver OHC for their patients, the logistic regression analysis showed that physicians working in the non-affluent regions (OR=2.9, 95% CI 1.36-6.15; p = 0.006) had greater willingness than did those from affluent regions.

## Discussion

Physicians establish early relationships with young children and their parents, and represent a trustworthy source of preventive information from birth. Therefore, along with the new approaches of public health, oral health promotion should be integrated into the existing preventive programs implemented by medical professionals [23]. Our study showed a considerable lack of knowledge about OHC among the primary care physicians working in the public sector in Tehran, and this may act as a barrier to their OHC delivery. The physicians, however, clearly acknowledged the importance of OHC in their profession.

### Knowledge among physicians and continuous medical education

The present study showed a lack of oral health knowledge among physicians, because our study group answered correctly only 18 of 34 oral health-knowledge questions. This is similar to findings of studies in Saudi Arabia, Italy, Canada, and the USA [13,20-22,24,25]. Moreover, the physicians’ knowledge level was significantly lower in the pediatric domain than in the dental domain and medical practice-related themes such as drugs causing xerostomia and increasing risk for dental caries. Our findings reveal that for questions in the pediatric area, more than 60% of the physicians' answers were incorrect. These findings are similar to those in other investigations regarding physicians' knowledge about early childhood caries and infant oral health [22]. In Iran, as in many other countries, most young children are not examined by a dentist until they reach the age of three years, but before their first birthday they see primary-care providers, including physicians, about ten times for health screenings [26]. If physicians were sufficiently knowledgeable in this field, they could consult parents regarding feeding practices, oral home care (brushing/flossing), and fluorides very early in the child's life. Moreover, they could diagnose caries lesions in their first stages and refer the child to a dentist for preventive procedures or early treatment if necessary.

Physicians’ training provides an excellent basis for deepening their understanding of dental health and prevention. Results show that physicians do gain information on and experience in general dental health during their careers, but special knowledge like that of pediatric dentistry requires special training. The patient population drives learning, and physicians working more with children and families are more aware of OHC than are those with fewer patients, and those working with children at high risk for dental diseases are more motivated to play an active role in OHC prevention [13]. The fact that learning is driven by motivation supports the adjustment of continuous medical education (CME) toward needs emerging from everyday practice [27]. Motivated practicing physicians will be reading or talking to colleagues to meet the challenges in their patient population, but they may also be more willing to attend CME and more importantly, willing to change their practices [28]. This approach has been supported by adult learning theory [29] and by social learning theory [30], followed by practices in implementing medical guidelines and knowledge [31]. Physicians in this study showed rather high motivation for CME for OHC in all groups, especially those working in disadvantaged regions with children and families, which is in line with the findings of Ditto et al. (2010), who reported the majority of their physicians as being interested in obtaining further knowledge on oral health [32].

CME can be effective in producing both short-term and long-term knowledge gains, as shown by available studies carried out mainly in Western health-care systems revealing CME to reduce prominent knowledge deficits [33,34]. In this study, however, primary knowledge on dental health issues was relatively low, so priming their information on diseases and supporting new practices might produce a significant effect. In general, CME can support the acquisition and retention of attitudes and behaviors needed for better clinical outcomes [35]; thus it should be encountered in physicians’ everyday professional lives.

### Attitudes among physicians

The present physicians had generally positive attitudes toward OHC and believed that they should be more knowledgeable in this field. Among the physicians, 77% were willing to carry out preventive measures, which is comparable to the findings of Prakash et al. [22] where 80% of the participants were willing to perform oral health-promotion activities. Another study indicated, however, that although those physicians believed that they play an important role in promoting oral health, they are not confident about becoming involved in this field [13]. The majority of physicians were aware of their lack of information on oral health and reported being willing to receive more education. Di Giuseppe et al. [18] described those physicians more knowledgeable in oral health as being more likely to play a role in children’s oral health promotion. This shows how important it is for physicians to have sufficient knowledge before acting and how their attitudes may be influenced by knowledge improvement. Another positive motivating issue in our study group was that almost all of the participants believed that it is necessary for a physician to know about oral health and to examine all patients’ oral cavity during their routine practice. This finding is in line with the studies by Prakash et al. in which physicians considered their role as “very important” in promoting oral health of children [22] and that of Sabbagh et al. reporting the majority of pediatricians as examining the oral cavity during the patient visit, confirming the fact that medical professionals have the will to dig more and recommend OHC to their patients [21].

### Socio-economic status

Our study included DHCs both from affluent and from non-affluent parts of the city of Tehran. In non-affluent parts of the city, people cannot afford private care; consequently, access to private practice is more difficult [15]. The number of public health centers and the patient turn-over are therefore higher in the non-affluent regions. Our results showed that physicians working both in the public sector and in private practices had significantly better knowledge than did their colleagues working solely in the public sector. This may reveal the effect of the higher number of patients seen and their increased exposure to oral health problems, both of which may have motivated them to seek and obtain more oral health information. The effect of the higher number of patients may also be seen among older physicians shown to be more knowledgeable in the dental field than were their younger colleagues. This finding is in line with another study’s that reported physicians spending more time in direct patient care as having higher levels of OHC knowledge [18]. No gender difference in the knowledge scores was observed in the univariate analysis, while the multivariate analysis revealed, contrary to some other findings, that female physicians had a higher level of knowledge in the pediatric field [10,18]. This may be related to female physicians’ better communication with children compared to male physicians [36].

In the present study, physicians from non-affluent parts of the city who may have been treating children from high-risk groups were more willing to receive OHC information and to deliver OHC preventive measures, and they clearly believed that they should examine the oral cavity and teeth throughout their routine patient care. This finding may be related to awareness of disparities in children's oral health and of access to care as important oral health challenges in many countries [3,26]. Physicians from non-affluent regions are in frequent contact with children from disadvantaged families who may suffer from various dental diseases. Untreated dental diseases can have a substantial impact on children in low-income populations [37,38]. This fact might explain physicians' willingness to obtain more OHC education and carry out preventive activities. Our findings call for providing the primary care physicians with OHC training to re-educate them in oral health promotion especially among non-privileged populations.

### Primary care in Tehran

The organized public health system for delivering primary health care in Iran was established in the 1970s, and OHC was integrated into the countrywide primary health care network in 1997. Under the supervision of DHCs, about 60% of the public health centers have OHC departments. The existing oral health system is mainly focused on 6- to 12-year-olds by means of a school-based national program, and also focused on pregnant and nursing mothers [39]. Taking into account the extremely young population of the country and high prevalence of dental caries in those under six years old, a public health approach to reach this young population is essential and could be best achieved by applying the potential of non-dental staff in primary health care, including physicians.

### Strengths and limitations of the study

The high response rate of the present study increases its value. The cross-sectional design allows investigation of potential links between level of knowledge and attitude; causality clearance, however, requires longitudinal studies. Surveys with a self-administered questionnaire might also produce non-responses, misconceptions, and errors [40]; these should be small in our survey, since the respondents were highly educated health professionals. In addition, because we included no solely private physicians, generalization of the results to all physicians is not justified.

## Conclusions

Our study revealed primary care physicians’ lack of knowledge in OHC but their generally positive attitude and willingness to participate in OHC. These findings provide a valuable incentive for planning of a CME program in primary care and its further research.

## Abbreviations

DHC: District Health Center; OHC: Oral Health Care; CME: Continuous Medical Education.

## Competing interests

The authors declare that they have no competing interests.

## Authors’ contributions

The authors SR, SZM, KP, and JIV have contributed equally to this work by making substantial contributions to conception and design, acquisition of data, and analysis and interpretation of data and being involved in drafting the manuscript or revising it critically for important intellectual content. All authors read and approved the final manuscript.

## Pre-publication history

The pre-publication history for this paper can be accessed here:

http://www.biomedcentral.com/1471-2458/12/855/prepub
